# The clinical characteristic and prognostic factors of leptomeningeal metastasis in patients with non‐small‐cell lung cancer‐a retrospective study from one single cancer institute

**DOI:** 10.1002/cam4.2156

**Published:** 2019-04-16

**Authors:** Weiwei Yan, Wang Jing, Ning An, Yaru Tian, Dong Guo, Li Kong, Hui Zhu, Jinming Yu

**Affiliations:** ^1^ School of Medicine and Life Sciences University of Jinan‐Shandong Academy of Medical Sciences Jinan Shandong China; ^2^ Department of Radiation Oncology Shandong Cancer Hospital Affiliated to Shandong University, Shandong Academy of Medical Sciences Jinan China

**Keywords:** EGFR‐TKIs, leptomeningeal metastasis, non‐small‐cell lung cancer, survival, WBRT

## Abstract

**Background:**

Leptomeningeal metastasis (LM) is a detrimental complication of advanced non‐small‐cell lung cancer (NSCLC), and the optimal therapeutic approach for LM patients is in shortage. This retrospective study aimed to investigate the clinical features and prognostic factors of NSCLC patients with LM.

**Methods:**

We retrospectively reviewed the medical records of NSCLC patients with LM at the Shandong Cancer Hospital and Institute between July 2014 and March 2018. Identified cases had pathology‐proven NSCLC with either positive cerebrospinal fluid cytology or leptomeningeal enhancement by MRI.

**Results:**

One hundred and thirty‐six NSCLC patients (58 men, 78 women) with LM were enrolled in the retrospective study; median age was 55 years (range, 29‐89 years). Fifty‐one patients harbored EGFR mutations, ALK rearrangement was detected in 6 patients. Treatment for LM consisted of EGFR‐TKIs alone in 11 patients, whole brain radiotherapy (WBRT) alone in 19 patients, Chemotherapy (ChT) alone in 12 patients, EGFR‐TKIs plus WBRT in 30 patients, WBRT plus ChT in 25 patients, and EGFR‐TKIs plus ChT in 24 patients. The median progression‐free survival was 3.9 months (95% confidence interval [CI]: 3.178‐4.622), and the median overall survival (OS_LM_) was 9.8 months (95% CI:7.5‐12.1). Thirty patients who received WBRT plus EGFR‐TKIs achieved longer survival than those who only received WBRT (median 13.6 vs 8.8 months; *P* = 0.027), but did not add any survival benefit than those only received EGFR‐TKIs (median 13.6 vs 13.9 months; *P* = 0.352). A multivariate analysis indicated that KPS ≥ 80 (hazard ratio [HR] = 0.592, 95% CI:0.369‐0.95; *P* = 0.03) and EGFR‐TKIs (HR = 0.507, 95% CI:0.283‐0.908; *P* = 0.022) after LM diagnosis were independent favourable predictors of survival, whereas smoking (HR = 1.181, 95% CI:1.009‐3.246; *P* = 0.047) was an independent predictor of poor survival.

**Conclusions:**

Our results suggest that patients with good performance statuses, non‐smoking patients, and the administration of EGFR‐TKIs might improve clinical outcomes in NSCLC patients with LM.

## 1 INTRODUCTION

Leptomeningeal metastasis (LM), or leptomeningeal carcinomatosis, is a devastating metastatic complication of systemic cancer that arises from the spread of malignant cells spread to the leptomeninges (pia and arachnoid mater), subarachnoid space, and cerebrospinal fluid (CSF) compartments.^1^, ^2^, ^3^, ^4^, ^5^, ^6^ LM was diagnosed in appropriately 5% of patients with malignant tumors.^7^ However, the autopsy outcomes showed that the incidence of LM might be 20% or more.^8^ Non‐small‐cell lung cancer (NSCLC) is characterized by a high incidence of central nervous system metastasis, with approximately 3.8% of all NSCLC patients developing LM in the course of their disease, which is prevalent in patients harboring EGFR mutations (9.4%).^9^, ^10^ Recently, LM has become an increasingly common diagnosis, likely as a result of improved survival due to effective therapeutic regimens against primary tumors, as well as improved neuroimaging techniques capable of discovering even small sites of meningeal dissemination.^11^ The effective treatment modality of LM is in shortage, and the median survival time is only 3‐11 months.^6^, ^9^, ^12^ The role of EGFR‐TKIs, systemic Chemotherapy (ChT), whole brain radiotherapy (WBRT), intrathecal chemotherapy (ITC), and ventriculoperitoneal (VP)‐shunt remain controversial.^10^, ^13^, ^14^, ^15^, ^16^

To our knowledge, there currently exists only a few randomized clinical trials that convincingly demonstrate the survival benefits of a specific treatment modality for LM. Therefore, optimal treatment modalities for LM in NSCLC patients remains poorly defined. Herein, we collected the data of 136 patients with LM to evaluate clinical outcomes and identify prognostic factors of LM patients.

## 2 PATIENTS AND METHODS

### 2.1 Patients

NSCLC patients with cytologically or radiographically proven LM were collected at the Shandong Cancer Hospital and Institute between July 2014 and March 2018. All patients were pathologically proven to have NSCLC. A diagnosis of LM was defined as positive CSF cytology (malignant cells) and/or focal or diffuse enhancement of leptomeninges, cranial nerves, and spinal cord diagnosed by brain and spine MRI. The study protocol was approved by the institutional review board and ethics committee of the Shandong Cancer Hospital and Institute, and informed consent was obtained from each participant included in the present study.

### 2.2 Data collection

The medical records of these patients included their demographic data, clinical characteristics, tumor‐related features, treatment modalities, and clinical outcomes. Clinical characteristics included age, gender, smoking status, and KPS at LM diagnosis. Tumor‐related features comprised NSCLC histological types, EGFR/ALK mutation status, treatments before the diagnosis of LM (including EGFR‐TKIs and WBRT), the presence of prior or concurrent brain or spinal metastases at LM diagnosis, gadolinium‐enhanced MRI findings, CSF cytological results, date of LM diagnosis, and date of death or last follow‐up. All treatment modalities were recorded, including ChT, WBRT, EGFR‐TKIs, ITC, VP‐shunt, and best supportive care.

### 2.3 Statistical analysis

Progression‐free survival (PFS_LM_) was defined as the time from LM diagnosis to the time of disease progression or death. Overall survival (OS_LM_) was defined as the time from LM diagnosis to the time of death or last follow‐up. PFS_LM_ and OS_LM_ were estimated using the Kaplan‐Meier method, and the differences between the study groups were compared using the log‐rank test. Cox's proportional hazard model was used to estimate the hazard ratio (HR) and associated 95% confidence interval (CI) on univariable analysis for the analysis of independent prognostic factors affecting OS. All variables that were either statistically significant in the univariate analysis or deemed to be clinically important were included in the multivariable analysis. All tests were two‐sided, and a *P*‐value less than 0.05 was considered statistically significant in all analyses. Analyses were conducted by using the SPSS Statistics version 20 (IBM Corporation, NY).

## 3 RESULTS

### 3.1 Characteristics of patients

One hundred and thirty‐six patients (58 men, 78 women) who met the inclusion criteria with LM from NSCLC were enrolled in the study. The median age was 55 years (range, 29‐89 years). Most of the patients (91.2%) were diagnosed with adenocarcinoma, 2 with adenosquamous carcinoma, 7 with squamous cell carcinoma, and 3 with large cell carcinoma. The mutational status of EGFR was evaluated in 78 patients, 51 of those patients were confirmed to have EGFR mutations, including 12 patients who harbored the exon 19 deletion (del 19), 31 patients who had the exon 21 Leu858Arg mutation (L858R), one of the patients with an L858R point mutation also had a point mutation in exon 20 (T790M), one patient who had point mutations in exons 18 and 20 (T790M), and 7 patients who harbored other mutations. ALK rearrangement was detected in 6 patients. Before the diagnosis of LM, 4 patients (2.9%) received ALK‐inhibitors, 50 patients (36.8%) received at least one line of EGFR‐TKI therapy, and 27 patients (19.9%) underwent WBRT for brain metastases (BMs). In the 51 LM patients harboring EGFR mutations, 30 patients were treated with EGFR‐TKI treatment before the diagnosis of LM. The clinical characteristics of the patients are detailed in Table 1.

**Table 1:** Patients’ characteristics (n = 136)

	No. of patients (%)
Age at the time of LM diagnosis (years)	
Median (range)	55 (29‐89)
<60	89 (65.4)
≥60	47 (34.6)
KPS at the time of LM diagnosis	
Median (range)	80 (40‐100)
≥80	106 (77.9)
<80	30 (22.1)
Gender	
Male	58 (42.6)
Female	78 (57.4)
Histologic subtype	
Adenocarcinoma	124 (91.2)
Non‐adenocarcinoma	12 (8.8)
Adenosquamous carcinoma	2 (1.5)
Squamous cell carcinoma	7 (5.1)
Large cell carcinoma	3 (2.2)
Smoking status	
Current/former smoker	37 (27.2)
Non‐smoker	99 (72.8)
EGFR gene mutation	
Presence	51 (37.5)
Absence	27 (19.9)
Unknown	58 (42.6)
ALK mutation	
Presence	6 (4.4)
Absence	24 (17.6)
Unknown	106 (78)
Previous EGFR‐TKI therapy before LM diagnosis	50 (36.8)
Previous WBRT before LM diagnosis	27 (19.9)

Abbreviations: ALK, anaplastic lymphoma kinase; EGFR, epidermal growth factor receptor; KPS, Karnofsky performance status; LM, leptomeningeal metastasis; NSCLC, non‐small‐cell lung cancer; TKI, tyrosine kinase inhibitor; WBRT, whole brain radiotherapy.

### 3.2 Patterns and clinical presentation OF LM

A total of 118 patients underwent a lumbar puncture at LM diagnosis, and 80 patients displayed malignant cells in CSF. All the patients showed typical findings in MRI of the entire neuraxis (the whole spine and brain), of which 120 were in the brain, 2 in the spine, and 14 in both. The diagnosis of LM was established by MRI alone in 56 patients (41.2%), and by both MRI and CSF cytology in 80 patients (58.8%). LM was detected in 24 patients (17.6%) at the initial diagnosis of NSCLC, and 112 patients (82.4%) developed LM in the course of disease. One hundred and fifteen patients (84.6%) were diagnosed with both LM and BMs; prior BMs were noted in 58 patients, of whom 27 had received prior WBRT. Fifty‐four patients were diagnosed with concurrent LM and BMs, and three patients developed BMs after LM diagnosis. The response to treatments of an extracranial disease at the time of LM diagnosis was a partial response and stable disease in 97 patients (71.3%) and progressive disease in 39 patients (28.7%) (Table 2).

**Table 2:** Patterns and clinical presentations of LM (n = 136)

	No. of patients (%)
LM with brain metastases	
Brain metastases before LM	58 (42.6)
Concurrent LM and brain metastases	54 (39.7)
Brain metastases after LM	3 (2.2)
LM only	21 (15.5)
Presentation of LM	
At the initial diagnosis of NSCLC	24 (17.6)
During treatment	112 (82.4)
Status of extracranial disease at LM diagnosis	
PR	21 (15.4)
SD	76 (55.9)
PD	39 (28.7)
The modality of LM diagnosis	
MRI alone	56 (41.2)
MRI+/cytology+	80 (58.8)

Abbreviations: LM, leptomeningeal metastasis; MRI, magnetic resonance imaging; NSCLC, non‐small‐cell lung cancer; PR, partial response; SD, stable disease; PD, progressive disease.

### 3.3 Treatments

The treatment modalities are summarized as follows: EGFR‐TKIs alone in 11 patients (8.1%), WBRT alone in 19 patients (14%), and ChT alone in 12 patients (8.8%). Thirty patients (22.1%) received EGFR‐TKIs and WBRT combinations, 25 patients (18.4%) underwent both WBRT and ChT, and 24 patients (17.6%) received EGFR‐TKIs and ChT. Twenty patients (14.7%) underwent ITC via lumbar puncture or ventricular reservoir. VP‐shunt operations were performed in 12 patients (14.6%) for palliation of hydrocephalus. Seventeen patients (12.5%) only received the best supportive care, and six patients received ALK‐inhibitors. More detailed results are presented in Table 3.

**Table 3:** Treatments after the diagnosis of LM

	No. of patients (%)
Chemotherapy only	12 (8.8)
EGFR‐TKIs only	11 (8.1)
WBRT only	19 (14)
ChT+EGFR‐TKIs	24 (17.6)
ChT+WBRT	25 (18.4)
EGFR‐TKIs+WBRT	30 (22.1)
ITC	20 (14.7)
Best supportive care	17 (12.5)
VP shunt operation	12 (8.8)
ALK‐inhibitors	6 (4.4)

Abbreviations: ALK, anaplastic lymphoma kinase; ChT, chemotherapy; EGFR, epidermal growth factor receptor; ITC, intrathecal chemotherapy; TKI, tyrosine kinase inhibitor; VP, ventriculoperitoneal; WBRT, whole brain radiotherapy.

### 3.4 Survival and prognostic factors

**Figure 1:**
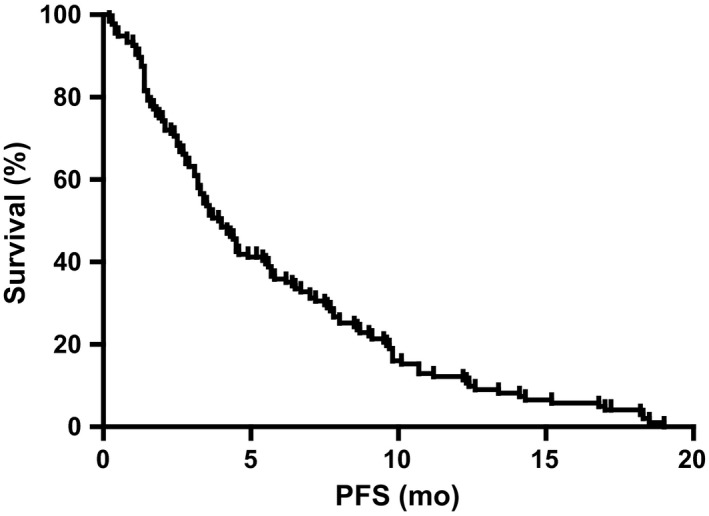
Progression‐free survival after the diagnosis of LM

**Figure 2:**
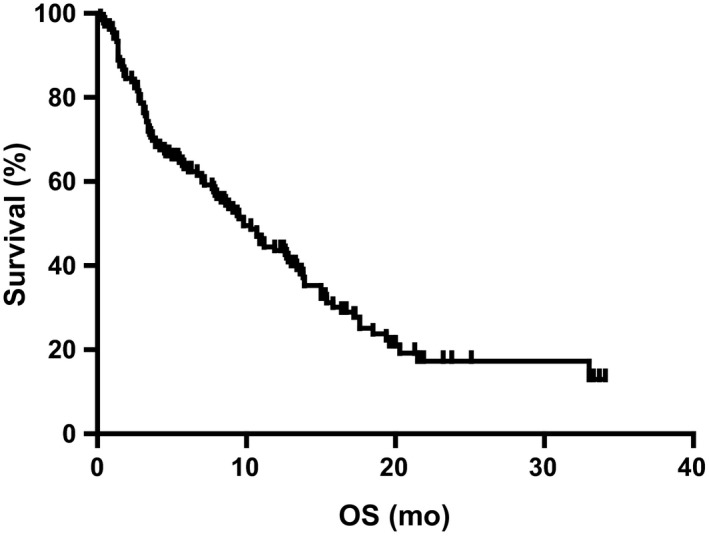
Overall survival after the diagnosis of LM

**Figure 3:**
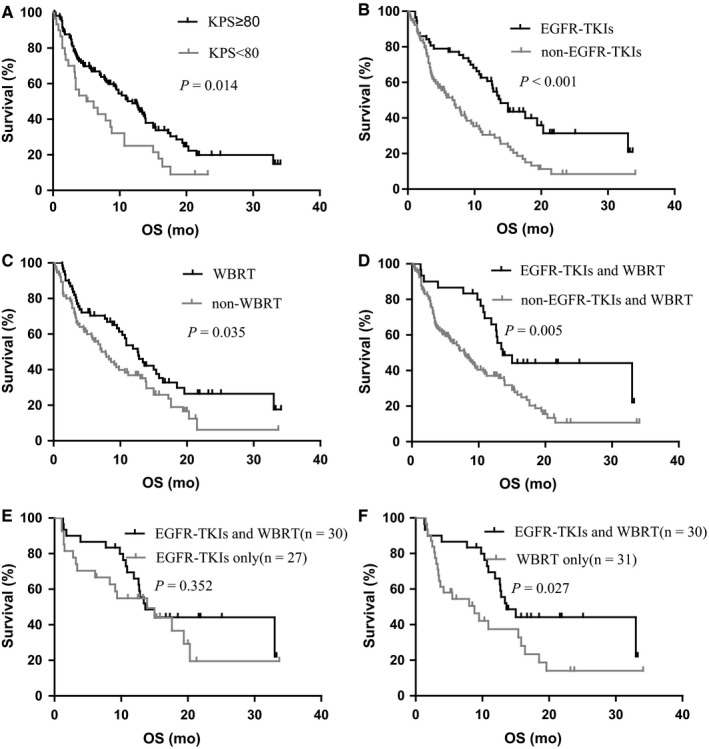
The Kaplan‐Meier analysis showing the overall survival of all patients. A, KPS ≥ 80 or KPS < 80 at the diagnosis of LM; B, received or did not receive EGFR‐TKIs; C, received or did not receive WBRT; D, received or did not receive WBRT plus EGFR‐TKIs; E, received EGFR‐TKIs plus WBRT or received EGFR‐TKIs only; F, received EGFR‐TKIs plus WBRT or received WBRT only

Follow‐up was completed in all 136 patients until June 2018. At the end of follow‐up, 38 patients (27.9%) were still alive, whereas 98 patients (72.1%) had died at last follow‐up. In the whole cohort, the median PFS_LM_ was 3.9 months (95% CI:3.178‐4.622; Figure 1), and the median OS_LM_ was 9.8 months (95% CI:7.5‐12.1; Figure 2). All relevant clinical factors that may be useful in predicting survival after LM diagnosis were evaluated in a univariate analysis using a log‐rank test. In univariate analysis of the entire cohort, male (*P* = 0.044), poor performance status (KPS < 80) (*P* = 0.014), smoking (*P* = 0.005), and lung adenocarcinoma (*P* = 0.024) predicted poor survival. In contrast, the application of WBRT (*P* = 0.035), EGFR‐TKIs (*P* < 0.001), and concomitant WBRT and EGFR‐TKIs (*P* = 0.005) predicted favorable survival. The patients with good performance statuses (KPS ≥ 80) acquired a prolonged survival period than those with a KPS less than 80 (median 11.9 vs 5.0 months, *P* = 0.014; Figure 3A). Fifty‐seven patients who received the EGFR‐TKI therapy after LM diagnosis demonstrated longer OS than those who without EGFR‐TKIs (median 13.9 vs 7.0 months; *P* < 0.001; Figure 3B). The median OS in 61 patients who received WBRT was longer than that of the patients who did not receive WBRT (12.6 vs 7.8 months; *P* = 0.035; Figure 3C). Furthermore, 30 patients who underwent both WBRT and EGFR‐TKIs showed more extended survival periods than those who did not follow the combined regime (median 13.6 vs 7.9 months; *P* = 0.005; Figure 3D). However, the survival period of these 30 patients was not more extended than that of those who only received EGFR‐TKIs (median 13.6 vs 13.9 months; *P* = 0.352; Figure 3E), although it was longer than that of those who only received WBRT (median 13.6 vs 8.8 months; *P* = 0.027; Figure 3F). The time of diagnosis of LM was an important factor affecting OS, although it was not significant. The median OS in 24 patients with LM at the time of initial diagnosis of NSCLC was longer than that of those who were diagnosed with LM during the treatment of NSCLC (median 13.9 vs 9.1 months; *P* = 0.076). ITC, VP‐shunt operation, ChT, the combination of WBRT and ChT, and EGFR‐TKIs plus ChT had no significant influence on the OS_LM_ of patients.

Multivariate analysis indicated that KPS ≥ 80 (HR = 0.592, 95% CI: 0.369‐0.95; *P* = 0.03) and the application of EGFR‐TKIs (HR = 0.507, 95% CI:0.283‐0.908; *P* = 0.022) were statistically significant factors associated with a favorable survival, whereas smoking (HR = 1.181, 95% CI: 1.009‐3.246; *P* = 0.047) was an independent predictor of poor survival. More statistical results are presented in Table 4.

**Table 4:** Univariate and multivariate analyses of the prognostic factors associated with survival in the entire cohort (n = 136)

	Univariate HR (95% CI)	P	Multivariate HR (95% CI)	P
LM present at the initial diagnosis of NSCLC (yes vs no)	0.584 (0.32‐1.07)	0.076		
Age (≥60 vs <60 y)	1.355 (0.9‐2.03)	0.138		
Gender (female vs male)	0.666 (0.45‐0.99)	0.044		
KPS at LM diagnosis (≥80 vs <80)	0.575 (0.37‐0.90)	0.014	0.592 (0.369‐0.950)	0.030
Smoking (current or former smoker vs non‐smoker)	1.816 (1.19‐2.78)	0.005	1.810 (1.009‐3.246)	0.047
Histologic subtype (adenocarcinoma vs non‐adenocarcinoma)	2.108 (1.08‐4.11)	0.024		
Treatment for LM				
EGFR TKI	0.481 (0.32‐0.73)	0.000	0.507 (0.283‐0.908)	0.022
WBRT	0.650 (0.43‐0.98)	0.035		
ITC	1.086 (0.63‐1.89)	0.768		
ChT	0.995 (0.67‐1.49)	0.980		
VP‐shunt operation	1.102 (0.57‐2.12)	0.769		
EGFR‐TKIS + WBRT	0.481 (0.28‐0.82)	0.005		
WBRT + ChT	0.911 (0.54‐1.54)	0.727		
EGFR‐TKIS + ChT	0.741 (0.43‐1.27)	0.270		

Abbreviations: ChT, chemotherapy; EGFR, epidermal growth factor receptor; ITC, intrathecal chemotherapy; KPS, Karnofsky performance status; LM, Leptomeningeal metastasis; NSCLC, non‐small‐cell lung cancer; TKI, tyrosine kinase inhibitor; VP, ventriculoperitoneal; WBRT, whole brain radiotherapy.

## 4 DISCUSSION

This retrospective study focused solely on NSCLC patients with LM. Compared to previous studies, this is a relatively large study conducted on the LM of NSCLC patients. In the present study, the median OS after the diagnosis of LM was 9.8 months, which was longer than that in almost all previous studies^9^, ^10^, ^15^, ^17^, ^18^; this may be due to a majority of our patients having good performance statuses and receiving frequent follow‐ups, as well as the early identification of LM by MRI. The development of molecularly targeted therapy also played an important role. In our study, 24 patients (17.6%) presented with LM at the time of the initial NSCLC diagnosis, with a particularly long median survival of 13.9 months, undoubtedly because these patients were better prepared to accept effective treatment than those who developed LM after one or more lines of therapy.

LM was much more frequent in NSCLC patients harboring EGFR mutations (9.4%).^9^ There is no doubt that EGFR‐TKI therapy is the standard first‐line treatment for NSCLC patients with EGFR mutations. Nevertheless, there is no standard regimen for treating LM because this population is excluded from randomized clinical trials. In the application of systemic treatment, including systemic ChT and EGFR‐TKIs, only EGFR‐TKIs was identified as an independent prognostic factor in the current analysis, which was consistent with findings from several previous studies.^10^, ^13^, ^14^, ^16^, ^17^ EGFR‐TKI therapy could control both leptomeningeal metastases and other lesions. In present study, the median OS of the 57 patients who received EGFR‐TKIs was prolonged, amounting to 13.9 months. Furthermore, among the 51 patients who harbored EGFR mutations, the median OS of the 36 patients who received EGFR‐TKIs was 15 months, which was significantly longer than the 4.5 months of the 15 patients who did not receive EGFR‐TKIs (*P* = 0.002). Liao et al^10^ recently reported that unselected patients who received EGFR‐TKIs for LM showed longer OS than those who did not (median 9.5 vs 1.7 months, *P* < 0.001), and the survival period in patients with EGFR mutations who underwent EGFR‐TKIs was significantly longer than that of patients who did not undergo the treatment (median 10.9 vs 2.3 months, *P* < 0.001); the findings of our current study are consistent with this report. All these promising results accordantly demonstrate the efficacy of EGFR‐TKIs on LM, especially in patients with sensitive EGFR mutations. Several retrospective analyses haved revealed that ITC increases the OS_LM_ of LM.^13^, ^14^, ^19^ In this study, only 20 patients received ITC, and 12 patients underwent VP‐shunt operation, with the absence of any significant impact on survival possibly due to the relatively small participating number.

Although WBRT may play a role in relieving neurological symptoms, there is a lack of evidence supporting its survival benefits, and its efficacy remains controversial.^6^, ^10^, ^14^, ^17^ In this study, 44.9% of the patients underwent WBRT for LM, and we indicated that WBRT was not an independent predictor of prolonged survival. This result is consistent with a previous study, Morris et al^14^ reported that there was no significant difference in median OS between patients who received WBRT and those who did not (*P* = 0.84). Nevertheless, in a retrospective cohort of NSCLC patients with LM, the median OS for patients who received WBRT for LM was longer compared with those who did not (10.9 vs 2.4 months, *P* = 0.002).^10^ LM is a neuraxis disease, with carcinoma cells dynamically circulating through all the compartments in a way that requires the complete irradiation of the craniospinal axis.^9^, ^20^, ^21^ However, total craniospinal irradiation is rarely recommended in the treatment of LM due to its substantial myelotoxicity.^20^ Therefore, the treatment of only a single CSF compartment with WBRT is used commonly in the management of LM, which may account for the smaller benefit of WBRT. In the present study, the combination of EGFR‐TKIs and WBRT did not increase any survival benefit compared with patients who only underwent EGFR‐TKIs (median 13.6 vs 13.9 months; *P* = 0.352). This result is consistent with a previous study,^9^ which reported that 33 patients treated with both WBRT and TKIs did not survive longer than those who only received EGFR‐TKIs (median 9.7 vs 10.1 months; *P* = 0.778). The role of WBRT is limited in the treatment of LM, and the combination with EGFR‐TKIs was not a beneficial treatment option.

We identified two treatment‐independent favorable prognostic factors, non‐smokers and KPS ≥ 80 at the diagnosis of LM. As reported in several retrospective analyses, better performance status is a well‐known favorable predictor of LM in patients.^5^, ^10^, ^13^, ^24^ In our current study, a majority of patients (77.9%) had high‐performance statuses, which exposed them to more opportunities to receive and benefit from radical treatments and acquire significantly more extended survival periods than those with poor performance statuses. To the best of our knowledge, few studies have reported the impact of smoking on the survival of LM patients. We identified smoking was an independent predictor of poor survival; the median OS for non‐smokers was longer than that of current or former smokers (12.7 vs 6.7 months, *P* = 0.005). Age and gender have been considered to be prognostic factors for survival in previous studies,^9^, ^19^, ^23^, ^25^, ^26^ but this was not the case in our study as we registered no significant difference.

Our research has several limitations. Firstly, our study is a retrospective study and there may be a selectivity bias. The patients we included in our study were relatively young, predominantly non‐smoking female, and primarily adenocarcinoma histology, which is a population with a high probability of harboring EGFR mutations and the dominant population for EGFR‐TKI treatment. Our results cannot be applied directly to patients with different populations and characteristics. Secondly, we used median PFS_LM_ and OS_LM_to evaluate the clinical survival benefits and did not consider the impact of previous treatments before LM diagnosis on survival. Moreover, patients may survive longer because of the effective control of extracranial disease and BMs with different therapies. The exact effects of a specific treatment on LM control cannot be identified in the present study. Hence, because of the retrospective nature of the current study, a cautious interpretation of the findings is necessary. This study was a single center, non‐randomized retrospective study. Therefore, the results need to be further validated in larger cohorts and prospective studies.

## 5 CONCLUSION

In conclusion, KPS ≥ 80 at the diagnosis of LM, non‐smokers, and EGFR‐TKIs lead to better clinical outcomes for NSCLC patients with LM.

## CONFLICT OF INTEREST

No potential conflicts of interest to revealed.

## ETHICS STATEMENT

All the patients enrolled in this study signed informed consents. The study was approved by the institutional review board and ethics committee of the Shandong Cancer Hospital and Institute.
